# Kenaf (*Hibiscus cannabinus* L.) Leaves and Seed as a Potential Source of the Bioactive Compounds: Effects of Various Extraction Solvents on Biological Properties

**DOI:** 10.3390/life10100223

**Published:** 2020-09-28

**Authors:** Md. Adnan, Ki Kwang Oh, Md Obyedul Kalam Azad, Myung Hwan Shin, Myeong-Hyeon Wang, Dong Ha Cho

**Affiliations:** 1 Department of Bio-Health Technology, College of Biomedical Sciences, Kangwon National University, Chuncheon 24341, Korea; mdadnan1991.pharma@gmail.com (M.A.); nivirna07@kangwon.ac.kr (K.K.O.); azadokalam@gmail.com (M.O.K.A.); 2 SJ Global Ltd., Gyeongsangnam do, Busan 635-890, Korea; dsd78@naver.com; 3 Department of Medical Biotechnology, College of Biomedical Sciences, Kangwon National University, Chuncheon 24341, Korea

**Keywords:** Kenaf leaves and seed, solvent extractions, GC-MS, antioxidant, cytotoxicity and anti-lung cancer, and antibacterial

## Abstract

*Hibiscus cannabinus* (Kenaf) is a potential source of bioactive constituents and natural antioxidant. The current study determined the impact of various solvents on extraction yield, recovery of polyphenol and flavonoid, antioxidant, anticancer, and antibacterial properties of Kenaf leaves and seed. The powder of leaves and seed was separately extracted with *n*-hexane, ethyl acetate, ethanol, and water solvent. Among them, the ethanol extract of leaves and seed showed the highest extraction yield, and their GC-MS analysis revealed a total of 55 and 14 bioactive compounds, respectively. The total polyphenols (TP) and flavonoids (TF) content were quantified by a spectrophotometric technique where water extracts displayed a noteworthy amount of TP and TF content compared to other extracts. A similar demonstration was noticed in antioxidant activity, evaluated by DPPH (2,2-diphenyl-1-picrylhydrazyl) and hydrogen peroxide scavenging capacity. In addition, cytotoxicity and anti-lung cancer activity were identified against mouse embryonic fibroblast (NIH3T3) and human lung cancer (A549) cells. All extracts of leaves and seed were observed as non-toxic to the NIH3T3 cells, but slight toxicity was expressed by *n*-hexane extracts at the optimum dose (1000 µg/mL) of treatment. In parallel, *n*-hexane and ethanol extracts (leaves and seed) exposed promising anti-lung cancer activity at the same concentration. Furthermore, antibacterial activity was assessed using disc diffusion assay, and seed extracts exhibited a significant inhibition zone against Gram-positive and Gram-negative microorganisms. Overall, Kenaf seed extracted with polar solvents was found very potent in terms of important bioactive compounds and pharmacological aspects, which can be an excellent biological matrix of natural antioxidants.

## 1. Introduction

One of the common manifestations of living cells is the generation of harmful pro-oxidants and reactive oxygen species (ROS), produced either due to the biological dysfunctions or as a result of cellular metabolisms (byproducts) [1]. Such free radicals may induce oxidative damage to proteins, lipids, and nucleic acids and lead to several life-threatening conditions, including cancer, neurodegenerative disease, ischemic heart disease, diabetes mellitus, and other chronic diseases [2,3]. In regards to preventing oxidative stress, antioxidants with adequate scavenging capacity have been used for appropriate balancing through regulation of oxidation or auto-oxidation processes. Commonly, many synthetic antioxidants, including butylated hydroxytoluene (BHT), tertiary butyl hydroquinone (TBHQ), and butylated hydroxyanisole (BHA) are widely added during food processing, preservation, as well as when preventing deterioration of color and texture [4]. However, these antioxidants have emerged as hazardous for human health in recent times [5]. Hence, researchers have refocused on alternative sources of antioxidants, especially from plant-derived products, which are very common in traditional medicine. Since plants contain heterogeneous metabolites and biomolecules in their compositions, with potential defense mechanisms, their synergistic action in treating various chronic diseases is an area of intense interest [6,7]. Therefore, investigation of valuable plants that possess abundant phenolics and other bio-active compounds must be screened meticulously in search for novel bioactive and safe antioxidants.

Cancer has 36 different types, and is a major burden for the world. In 2018, 18.1 million of new cases and 9.6 million deaths were recorded, where collateral, liver, lung, thyroid, and stomach cancer were highly prevalent in both men and women [8]. By 2030, it is foretold that the new suspected cases and death toll by cancer will surpass around 26 million and 17 million, respectively [9]. Hence, researchers are searching for an alternative method using novel chemotherapeutic agents, which are most importantly safe, non-toxic, and easily available. In this regard, medicinal plants, traditional medicine, phytomedicine, and the pharmacological potential of plant compounds are the best choice. Currently, 60% of approved anticancer drugs have been derived from a natural source [10]. Besides, polyphenols and antioxidant-rich fruits and vegetables can also play a pivotal role in malignancy transformation and cancer development [11]. Analogues to cancer, bacterial resistance to all classes of antibiotics are one of the most alarming issues for global public health. In recent decades, no significant antibiotics have been discovered which urge the development of new drugs with novel mechanisms of action against various pathogens [12]. Due to the high chemical diversity, bioactive compounds and metabolites extracted from natural products are considered as viable candidates for bioprospecting programs, which can intervene in a range of microbial pathways [13]. 

Kenaf (*Hibiscus cannabinus*) is an annual herbaceous dicotyledonous plant, belongs to the *Malvaceae* family, is widely distributed in Asia and Africa, and grows mostly in temperate to tropical areas [8]. Kenaf (leaves and seed) has many significant medicinal properties, including anticancer, antioxidants, analgesic, anti-inflammatory, aphrodisiacs, and hepatoprotective activities [9,10]. In traditional medicine, Kenaf is used to treat various diseases; for instance, a paste of the leaf and stem is used to treat Guinea worms disease and anemia in Africa [10]. Moreover, in ayurvedic medicine, the leaves are used to treat various disorders, such as of the blood, diabetes, bilious, the throat, and coughs [10,11]. Furthermore, flower juice and seed are consumed for biliousness and bruises [12]. These medicinal benefits are exposed due to the presence of abundant phenylpropanoid compounds in the Kenaf plant [13]. Besides, many bioactive compounds, such as omega-3 fatty acids and sterols [11,14], as well as phenolic compounds, including kaempferol, vanillic acid, syringic acid, caffeic acid, gallic acid, p-hydroxybenzoic acid, p-coumaric, and ferulic acid have been identified earlier from seed extracts [12]. Previously, Kenaf seed was used to prepare biopolymer-mediated nanocomposites to enhance seed flour’s antioxidant capacity [5]. In addition, efficient silver nanoparticles were synthesized using the seed extract, which manifested promising antibacterial and anticancer activities [15].

However, despite having such important biological properties of different parts of this plant, a few scientific reports have been found based on its pharmacological aspect. Hence, in this study, we aimed to identify the content of secondary metabolites, antioxidant, anticancer, and antibacterial properties of Kenaf leaves and seed. To evaluate this, various solvents were used to extract effective phytoconstituents in a particular solvent. Besides, ethanol extract of both leaves and seed were explored with the aid of gas chromatography-mass spectrometry (GC–MS) analysis. In particular, this research demonstrates the influence of various solvent extracts on the Kenaf leaves and seed, and also reveals their pharmacological activities. 

## 2. Method

### 2.1. Chemicals

Ethanol, Ethyl acetate, *n* hexane, phenolic reagent (Folin-Ciocalteu, 2N), sodium carbonate (Na_2_CO_3_), aluminum nitrate (AlNO_3_), potassium acetate (CH_3_COOK), DPPH (2,2-diphenyl-1-picrylhydrazyl), hydrogen peroxide (H_2_O_2_), Mueller Hinton agar media, and ampicillin were procured from Sigma (Sigma Chemical Co., St. Louis, MO, USA). Other chemicals, such as a water-soluble tetrazolium (WST) assay kit (EZ-Cytox, Daeil Lab Service, Gwangiu, Korea), PBS, Dulbecco’s modified eagle medium (DMEM), fetal bovine serum (FBS), Roswell Park Memorial Institute (RPMI) medium, and penicillin-streptomycin (PS) were purchased from Gibco (Waltham, MA, USA) and Thermo Fishers Scientific (Seoul, Korea). The NIH3T3 A549 cells were collected from the Korean Cell Line Bank (KCLB, Seoul, Korea).

### 2.2. Plant Material and Extracts Preparation

The leaves (younger completely formed leaves) and seed (collected before harvesting) of Kenaf (Israeli verity) were supplied by the Kenaf Company (Gangwondaehak-gil, Chuncheon-si, Gangwon-do, Korea, 24341). The collected samples (leaves and seed) were cleaned and placed to be oven-dried for a week by maintaining a suitable temperature (55 °C). Afterwards, the dried samples (water content was zero) were pulverized into a coarse powder using a pin crusher (JIC-P10-2; Myungsung Machine, Seoul, Korea). The ground samples were passed through 300 µm mesh-size sieves to form a fine powder, and stored at room temperature before extract preparation.

The fine powders of Kenaf leaves (500 g) and seed (500 g) were soaked separately in 2.5 L of ethanol for five days at room temperature, with continuous stirring and shaking on a Rotary Shaker (JEIOTECH SI-900 R). Afterwards, the solvent extracts were first filtered by a sterilized cotton plug, and then we used Whatman filter paper No. 1, followed by evaporation through the rotary evaporator at 50 °C to get semisolids of Kenaf leaves (36 g) and seed extract (41 g). The same process was repeated for *n*-hexane, ethyl acetate, and water solvent extraction. After evaporation (above mentioned condition) of each solvent, the yield of *n*-hexane (leaves: 3.17 g; seed: 4.27 g), ethyl acetate (leaves: 6.17 g; seed: 10.76 g), and water (leaves: 8.12 g; seed: 9.29 g) extract were collected and preserved in a refrigerator for further investigation. In order to investigate the pharmacological potentials, the samples were prepared in 5% di-methyl sulfoxide (DMSO). 

The yield after extraction was calculated as follows:

Yield (%) = (Dried extract weight/Dried sample weight) × 100.

### 2.3. Gas Chromatography-Mass Spectroscopy (GC-MS) Analysis of Kenaf (Leaves and Seed) Extracts

The bioactive compounds of Kenaf leaves and seed (ethanol extract) were detected by GC-MS analysis using an Agilent Technologies 7890A capillary gas chromatograph, along with a mass spectrometer system. GC was equipped with a 30 m × 0.25 mm × 0.25 μm DB-5 capillary column. Initially, the instrument was maintained at a temperature of 100 °C for 2 min and 6 s. The temperature was risen to 300 °C at the rate of 25 °C/min and maintained for 20 min at the end of this period. The injection port temperature and the helium flow rate were ensured to be 250 °C and 1.5 mL/min, respectively. The ionization voltage was 70 eV. The sample was injected in split mode at 5:1. The MS scan range was set at 35–550 (*m*/*z*). The fragmentation patterns of mass spectra were compared with those stored in the using W8N05ST Library MS database. The percentage of each compound was calculated from the relative peak area of each compound in the chromatogram. The concept of integration used the Chem Station integrated algorithms. 

### 2.4. Quantitative Analysis 

#### 2.4.1. Total Phenolic Content (TPC)

The total content of phenol in Kenaf leaves and seed extracts was assessed following the method of Folin-Ciocalteau [16]. The Folin-Ciocalteau reagent (200 µL, 1 N) was added in the test tube containing 1 mL of the sample (10 mg/mL). The volume of the mixture was increased by the addition of deionized water (1.8 mL) and kept (3 min at room temperature) for the reaction after the vortex. Afterwards, 400 µL of sodium carbonate (10% *v*/*v*) was added to the reaction mixture. Finally, the volume was adjusted up to 4 mL by adding deionized water (600 µL). The mixture was placed in dark ambience for incubation (1 h at room temperature), and the test was done in triplicates. The absorbance was measured against the blank (water) at 725 nm by the spectrophotometer (UV-1800 240 V, Shimadzu corporation, Kyoto, Japan). The TPC was calculated from a calibration curve (plotting the value of absorbance vs. concentration) using gallic acid and expressed as mg of GAE (gallic acid equivalent) per 100 g of extract.

#### 2.4.2. Total Flavonoid Content (TFC)

The total flavonoid content in Kenaf leaves and seed extracts was determined according to the previously described method [17] with some modifications [18]. In brief, 0.5 mL aliquot of extract (10 mg/mL) was mixed with 100 µL of aluminum nitrate (10% *w*/*v*), 100 µL of potassium acetate (1M), and 3.3 mL of ethanol. The mixture was vortexed and placed for incubation (40 min at room temperature). TF content of the extracts was measured at 415 nm by the spectrophotometer (UV-1800 240 V, Shimadzu corporation, Kyoto, Japan) and expressed in mg/100 g QE (quercetin equivalent). 

### 2.5. Antioxidant Activity

#### DPPH Free Radical and Hydrogen Peroxide (H_2_O_2_) Scavenging Activity

The antioxidant activity of Kenaf leaves and seed extracts was evaluated using DPPH free radical and H_2_O_2_, following the method of Braca et al. [19] and Adnan et al. (2020) [5]. For the DPPH, 3 mL of freshly prepared DPPH (0.004% *w*/*v* in methanol) was added to the 0.5 mL of stock solution. The reaction mixture was vortexed and placed in the dark ambience for incubation (30 min at room temperature). For H_2_O_2_ scavenging, 0.6 mL of H_2_O_2_ solution (4 mM prepared with 0.1 M phosphate buffer pH 7.4) was mixed with 0.4 mL of stock solution and then incubated for 10 min. The scavenging of DPPH and H_2_O_2_ were measured at 517 nm and 230 nm, respectively by the spectrophotometer (UV-1800 240 V, Shimadzu Corporation, Kyoto, Japan). The percentage of scavenging capacity was calculated against negative control (methanol + DPPH) and (stock solution without H_2_O_2_) expressed by the following equation: Scavenging effect (%) = [(Abs_c_ − Abs_s_)/Abs_c_] × 100, where Abs_c_ is the absorbance of control; Abs_s_ is the absorbance of DPPH radical/H_2_O_2_ + sample (extract/standard).

### 2.6. Cell Culture, Cytotoxicity, and Anti-Lung Cancer Assay

The cytotoxicity and anti-lung cancer potentiality of various solvent extracts (leaves and seed) were evaluated against NIH3T3 and A549 cells, employing the WST assay kit. The NIH3T3 and A549 cells were cultured in the penicillin and streptomycin (PS)-incorporated DMEM and RPMI medium, respectively. The cells were incubated (CO_2_ incubator at 37 °C for 24 h) and the quality of cells (confluences and morphology) were observed under light microscopy. The prepared cells were further used in cytotoxicity and anti-lung cancer analysis. Briefly, the NIH3T3 (5 × 10^4^) and A549 (1 × 10^5^) cells were seeded in the 96-well plates (containing DMEM and RPMI medium, respectively) and incubated (CO_2_ incubator at 37 °C for 24 h) until they reached the desirable confluence (80–90%). The incubated cells were treated with the different concentrations (62.5, 125, 250, 500, and 1000 µg/mL) of extracts and incubated with a similar condition. Finally, Ex-CyTox reagent (10 µL) was added to each well and absorbance was recorded at 450 nm. As a negative control, 5% DMSO was used. From the obtained absorbance, the cytotoxicity and cell viability of the extracts were calculated according to the formula described elsewhere [15]. 

### 2.7. Antibacterial Activity 

Antibacterial activity was analyzed using the disc diffusion method [15]. In brief, Mueller-Hinton agar was prepared and placed into Petri dishes for solidification (under laminar airflow). Gram-positive *Staphylococcus aureus* (ATCC 6538), *Bacillus subtilis* (ATCC 6633), and *Bacillus cereus* (ATCC 14579) and Gram-negative *Salmonella Typhi* (ATCC 29629), *Pseudomonas aeruginosa* (ATCC 9027), and *Escherichia coli* (ATCC 8739) microorganisms were cultured overnight. Of each prepared bacteria, 100 µL (bacterial inocula were adjusted to 10^7^ CFU/mL) was spread smoothly on the agar surface, and then a sterile disc (8 mm diameter) was laid upon an agar plate (seeded). Each extract with desired concentration (50 mg/mL) was loaded on these discs and incubated (at 37 °C for 24 h). The zone of inhibition was recorded and measured in mm. The 5% DMSO was used as a negative control, and as a positive control, ampicillin (25 μg/mL) was used.

## 3. Statistical Analysis

All data were expressed as mean ± standard deviation (SD) of several measurements. The obtained results (total phenol, flavonoid, and antioxidant activity) were compared among the composition using a paired *t*-test in order to observe the significant differences at the level of 5%. The paired *t*-test between the mean values was analyzed by MINITAB (version 17.0, Minitab Inc., State College, PA, United States). Data of cytotoxicity was analyzed using GraphPad Prism 6.0 statistical software. Values are expressed as mean ± SD (*n* = 3) and two-way ANOVA, followed by the Bonferroni test. There was a significant difference when comparing each column to all other columns at * *p* < 0.05, ** *p* < 0.01, and *** *p* < 0.001. 

## 4. Results and Discussions

### 4.1. Extraction Yields

**Figure 1:**
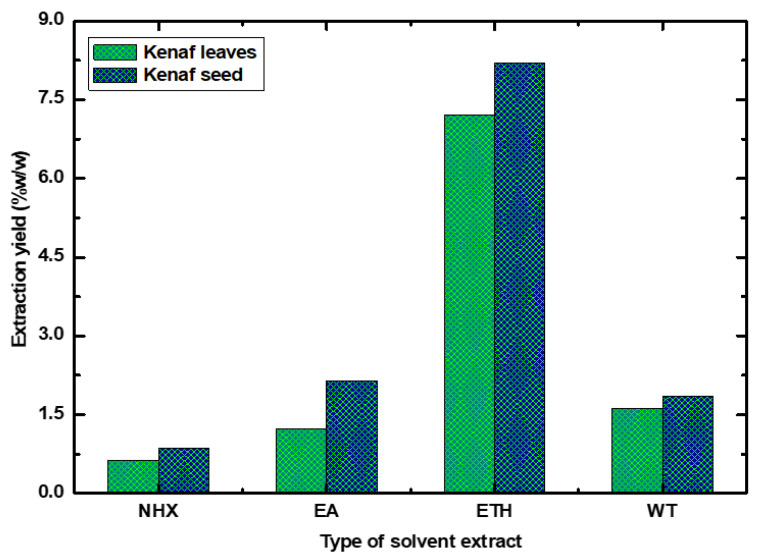
Effect of different solvents on the extraction yield.

Various parts of a plant occupy a pool of bioactive compounds containing potential chemical groups which consistently protect both plants and humans from cellular oxidative damage [20]. However, to explore all these major classes of chemicals, efficient extraction techniques, such as maceration, subcritical water extraction, soxhlet extraction, supercritical fluid extraction, and ultrasonic-assisted extraction with suitable solvents are a prerequisite [21]. Importantly, plant-derived phytoconstituents compose numerous chemical characteristics and polarities that are not readily soluble in a particular solvent [22]. Therefore, various solvents with differing polarities must be studied to extract potential bioactive compounds from the plant [23]. Our study used different solvent types to evaluate the impact of extraction yield, and here, the seed extracts exhibited a higher yield than the leaves’ extracts (Figure 1). In both cases, the highest and lowest extraction yields were recorded for the ethanol (ETH) and *n*-hexane (NHX) extracts, respectively. After the ethanol extract, the yield differences were observed in another solvent extraction, such as ethyl acetate (EA), followed by the water (WT) extract for the Kenaf seed, and water followed by an ethyl acetate extract for kenaf leaves. Sim et al. [24] reported that pulsed ultrasonic-assisted Kenaf leaves extraction with water showed the highest extraction yield compared to methanol, ethanol, and acetone solvent. Yusri et al. [25] also followed a similar extraction method for Kenaf seed, and the highest extraction yield was obtained for the hexane extract. However, in our study, the conventional extraction method was followed, and the observed variation of yield extraction might have been due to the influence of a heterogeneous solvent used with different polarities, and the results are in line with the previously reported extraction yields of rice bran [26] and many medicinal plants [27]. 

### 4.2. GC-MS Analysis of Ethanol Extract for Kenaf (Leaves and Seed) 

**Figure 2:**
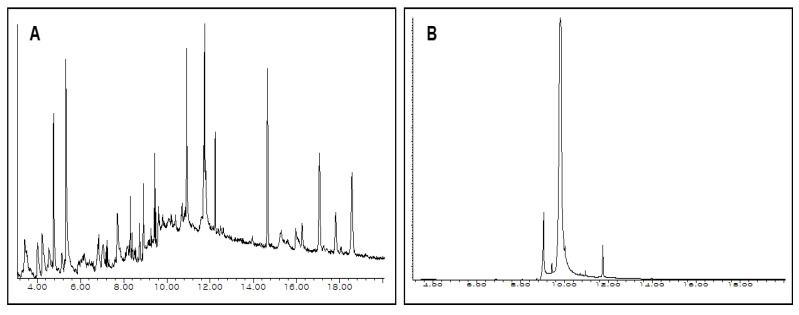
Total ionic chromatogram (TIC) of ethanol extract of Kenaf leaves (**A**) and seed (**B**) obtained by GC-MS, with an energy of ionization of 70 eV.

Since a higher yield was found for the ethanol extract of both Kenaf leaves and seed, we further conducted GC-MS analysis to reveal their chemical profile. It is well-known that GC-MS with specific detection systems is a valuable tool for separating and identifying the components from complex volatile mixtures. GC-MS consists of two analytical techniques where GC splits the component from the mixture, and MS analyzes each of the components separately [28]. In addition, compounds that are small, adequately volatile, and thermostable in the GC environment can be easily analyzed by GC–MS [29]. Previously, GC-MS analysis on the hexane extract of Kenaf leaves and seed manifested 13 and 10 phytoconstituents, respectively [12]. In our study, GC-MS analysis of Kenaf leaves and seed (ethanol extracts) revealed 55 and 14 compounds, having retention times from 3.42 to 18.56 min and 4.00 to 17.07 min, respectively, which are enlisted in Table 1, and their total ionic chromatograms (TIC) are depicted in Figure 2. Among the 55 compounds in Kenaf leaves, the following components considering their peak area are documented: 5-Hydroxymethylfurfural (7.20%), 2-Stearoylglycerol (5.44%), 1,5,9,13-Tetradecatetraene (4.50%), Vitamin E (4.45%), alpha-Amyrin (3.76%), 4H-Pyran-4-one, 2,3-dihydro-3,5-dihydroxy-6-methyl- (3.70%), Clionasterol (3.93%), 2-Linoleoylglycerol (3.40%), and Hexadecanoate (3.06%). Besides, the detected compounds from Kenaf seed are 9-octadecanoic acid (z)- (77.46%), Hexadecanoic acid (10.25%), 9, 12-octadecadienoic acid (z, z)-, 2-hydroxy-1-(hydroxymethyl)ethyl ester (6.21%), and Linoleic acid (4.43%).

### 4.3. Total Phenol and Flavonoid Content

**Figure 3:**
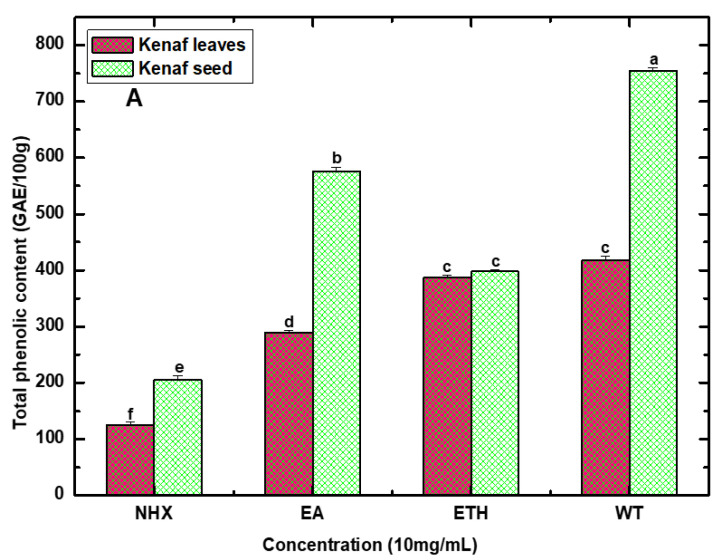

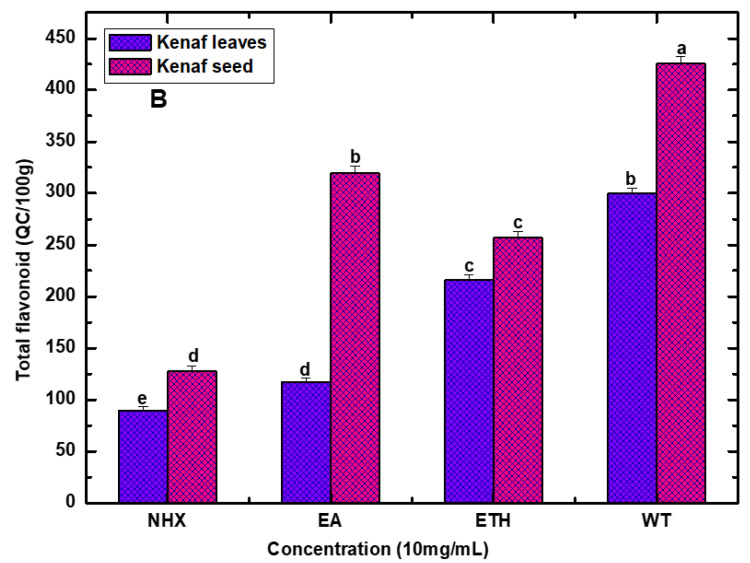
Total phenolic (**A**) and flavonoid (**B**) content of various solvent extracts (NHX, EA, ETH, and WT) of Kenaf leaves and seed. Values are expressed as mean ± SD (n = 3). Values marked by different letters in each column are significantly different by t-test (*p* < 0.05). GAE = gallic acid equivalent, QC = quercetin equivalent, NHX = *n*-Hexane extract, EA = Ethyl acetate extract, ETH = Ethanol extract, and WT = Water extract.

Four different extracts (NHX, EA, ETH, and WT) of Kenaf leaves and seed were analyzed to quantify phenolic and flavonoid content. The phenolic content was assessed by gallic acid and expressed as GAE per 100 g of dry extract (Figure 3A), whereas flavonoid content was estimated by quercetin and expressed as QE per 100 g of dry extract (Figure 3B). The highest amount of phenolic and flavonoid content was recorded in seed compared to leaves. Particularly, WT extract demonstrated the highest total phenol content for both Kenaf seed (754.6 ± 3.14 mg/100 g dry extract) and leaves (418.7 ± 3.47 mg/100 g dry extract). Similarly, the maximum flavonoid content was also detected from the WT (425.33 ± 4.39 and 299.17 ± 3.43 mg/100 g dry extract) extract of both Kenaf seed and leaves. However, the other solvent seed extracts of EA followed by ETH and NHX, as well as leaf extracts of ETH, followed by EA and NHX were noted as significant for both TPC and TFC.

The highest TPC and TFC observed in the water extract might have resulted from its high polarity index [31]. The important phytoconstituents of a plant can be polar or nonpolar in nature—mainly, phenolic compounds possess abundant hydroxyl groups, which are responsible for dissolving of the polar solvent [32]. In addition, the ethyl acetate extract displayed better extraction of TPC and TFC, which confirmed that this solvent is also efficient for extracting secondary metabolites. It is stated that the ethyl acetate solvent has potential for the extraction of phenolic compounds, and this statement is in agreement with the result found from the EA extract of Kenaf seed [33]. Besides, noteworthy TPC and TFC manifested by the ethanol extract might be owing to the presence of methyl radicles, which can easily conjugate with phenolic or flavonoid compounds and allow efficient solvation [34]. In contrast, hexane showed poor extraction of TPC and TFC, which could be attributed to the lower polarity or strong non-polar nature of the solvent [35].

### 4.4. Antioxidant Activity

**Figure 4:**
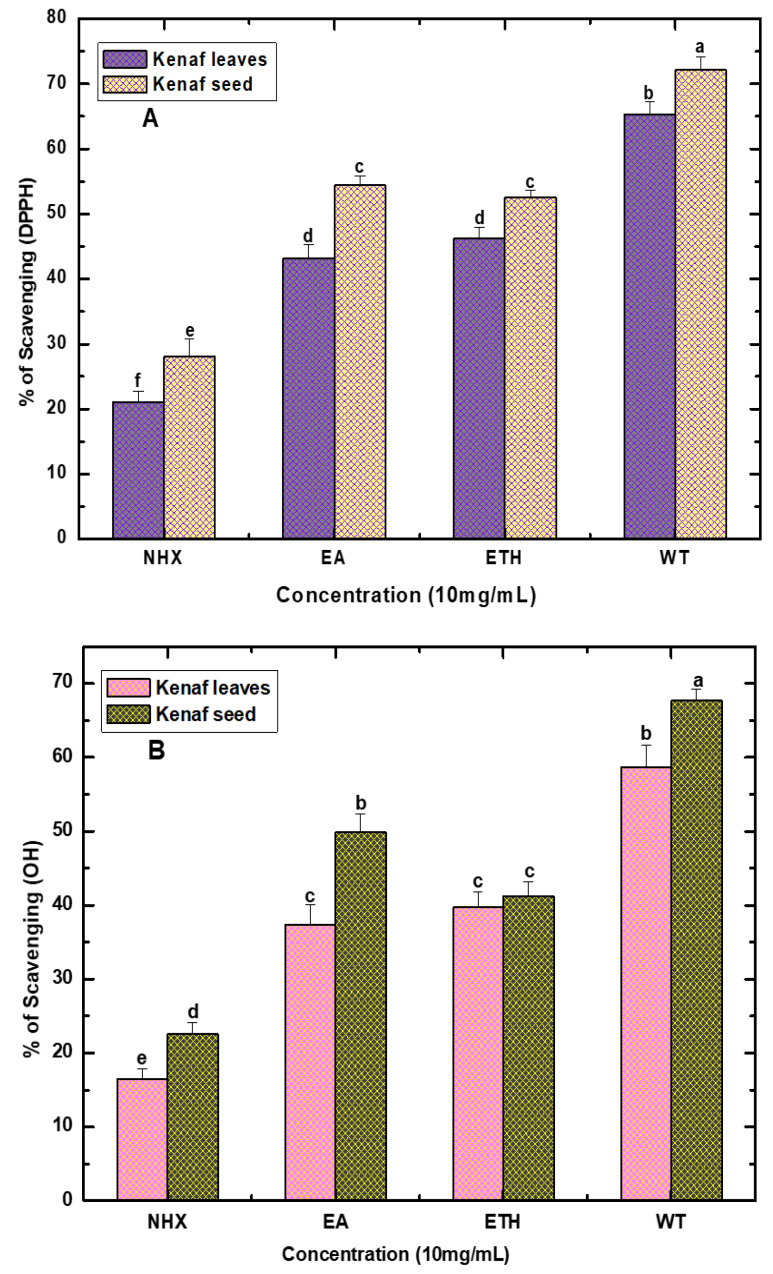
DPPH (**A**) and hydrogen peroxide (**B**) scavenging capacity of various solvent extracts (NHX, EA, ETH, and WT) of Kenaf leaves and seed. Values are expressed as mean ± SD (n = 3). Values marked by different letters in each column are significantly different by t-test (*p* < 0.05). NHX = *n*-Hexane extract, EA = Ethyl acetate extract, ETH = Ethanol extract, and WT = Water extract.

Figure 4A,B displays the percentage inhibition of the DPPH and OH free radical scavenging capacity of Kenaf leaves and seed extracts. The activity notably varied among the various extracts, and the overall higher percentage of scavenging capacity was observed in the seed extracts compared to leaves. In both cases, the antioxidant capacity of ethanol and ethyl acetate extract was almost identical; even no noticeable changes in regard to activity were noted for the DPPH and H_2_O_2_ free radical scavenging. However, in the case of DPPH, the highest antioxidant activity of Kenaf seed was recorded for WT (73.12 ± 2.06%), followed by ethyl acetate (54.45 ± 1.38%), ETH (52.49 ± 1.12%), and hexane (28.09 ± 2.61%) extract. However, Kenaf leaves also exhibited 65.35 ± 1.86% DPPH scavenging for the WT, 46.27 ± 1.71% (EHT), 43.19 ± 2.15% (EA), and 21.07 ± 1.65% (NHX) extracts. These findings were analogous with many previous studies, where authors concluded that relatively high polar solvent extracts, such as water, methanol, and ethanol exposed higher DPPH free radical potential than the non-polar solvent extracts. Likewise, DPPH, a similar pattern of antioxidant activity, was observed in the case of the H_2_O_2_ scavenging test. Here, Kenaf seed extracts demonstrated strong hydroxyl radical scavenging activity, ranging from 67.69 ± 1.46 to 26.41 ± 2.58%, whereas leaf extracts manifested between 53.86 ± 0.43 and 19.52 ± 1.83%.

Interestingly, an interplay relationship exists between antioxidant activity and the quantity of secondary metabolites [36]. Usually, phenolics are regarded as the predominant antioxidant components, and scavenging activities of these components are directly proportional to the total content of phenolics [18]. Hence, the observed higher antioxidant activity in our study might be attributed to secondary metabolites, such as higher content of polyphenol and flavonoids of the extracts. Nevertheless, these outcomes indicate that this plant has remarkable scavenging capacity.

### 4.5. Cytotoxicity and Anti-Lung Cancer Activity

**Figure 5:**
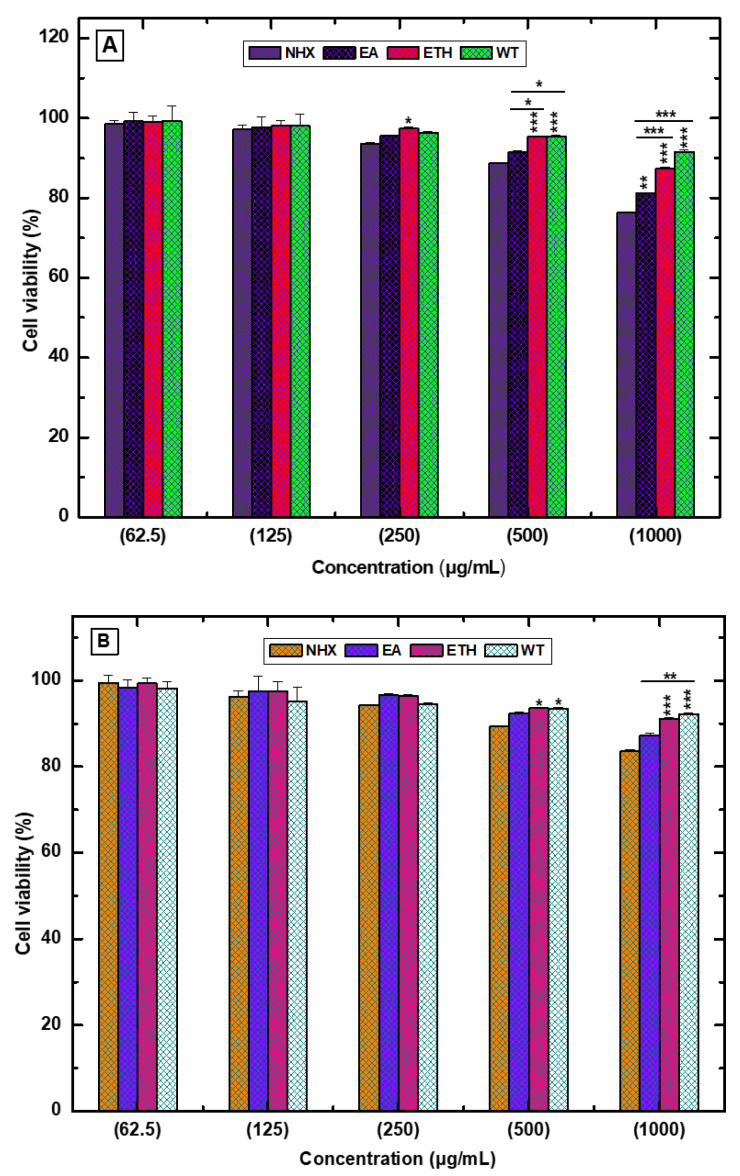
Cytotoxicity of various solvent extracts (NHX, EA, ETH, and WT) of Kenaf seed (**A**) and leaves (**B**). Here, NHX = *n*-Hexane extract, EA = Ethyl acetate extract, ETH = Ethanol extract, and WT = Water extract. Data was analyzed using GraphPad Prism 6.0 statistical software. Values are expressed as mean ± SD (n = 3), and a two-way ANOVA andBonferroni test were applied. They were significantly different when comparing each column to all other columns at * *p* < 0.05, ** *p* < 0.01, and *** *p* < 0.001.

The cytotoxicity of the Kenaf leaf and seed extracts was determined against NIH3T3 cells. Figure 5A displays the Kenaf seed extracts which expressed slight toxicity with increasing concentrations in a dose-dependent fashion. Notably, the hexane and ethyl acetate extracts showed higher cytotoxicity of23.64 ± 0.12% and 18.77 ± 0.13%, respectively, and cell growth inhibition was detected at a higher concentration (1000 µg/mL). In the case of Kenaf leaves (Figure 5B), a similar cytotoxicity pattern was also exposed by the hexane and ethyl acetate extracts. Here, cell growth inhibition was recorded as 16.37 ± 0.20% and 12.7 ± 0.37%, respectively, at the same concentration. However, treatment of water and ethanol extracts (both seed and leaves) did not reveal any obvious toxicity toward the NIH3T3 cells, even at the higher concentration treatment, which indicates that these extracts are comparatively safe than the others. The observed healthy cells after the treatment of water and ethanol extract might be caused by their higher content of polyphenol, flavonoid, and promising antioxidant activity, which acted as nutrients for the cell growth [37]. In contrast, the toxicity demonstrated by hexane extract might be attributed to its poor antioxidant capacity, which affected the cells by producing reactive oxygen species [38].

**Figure 6:**
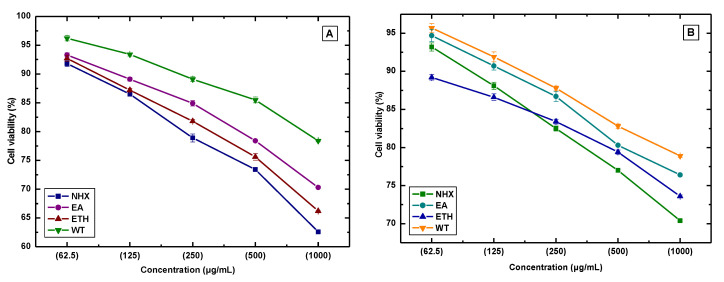
Anti-lung cancer activity of various solvent extracts (NHX, EA, ETH, and WT) of Kenaf seed (**A**) and leaves (**B**). Here, NHX = *n*-Hexane extract, EA = Ethyl acetate extract, ETH = Ethanol extract, and WT = Water extract. Values are expressed as mean ± SD (n = 3).

Figure 6A,B displays the anti-lung cancer activity of Kenaf seed and leaves against A549 cells. Here, NHX extract exposed the highest anti-lung cancer activity in a concentration-dependent response, where treatment of the optimal concentration (1000 µg/mL) revealed 37.4% cell death by Kenaf seed and 29.6% by Kenaf leaves. The comparable accord was also observed for ETH and EA extract. In Kenaf seed, cell viability was reduced to 33.8% by ETH, while EA decreased 29.7% cell viability at the highest concentration. In contrast, ETH (26.4%) and EA (23.6%) of Kenaf leaves also manifested moderate anticancer activity. Interestingly, NHX demonstrated toxicity against NIH3T3 cells, and ETH was found to be safe for NIH3T3 cells; however, during an anti-lung cancer test, both extracts exhibited remarkable inhibition of A549 cell growth, which concludes that the ETH extract is relatively safer than the other extracts. Previously, a study conducted by Wong et al. revealed that the optimum concentration of Kenaf seed extract strongly inhibited the cell growth of *HeLa* (CCL–2), breast cancer (MCF–7), colon cancer (HCT–116), and lung cancer (SK–LU1) [39], which also support our findings. Importantly, the treatment of 5% DMSO in the NIH3T3 and A549 cells did not expose any notable toxicity, wherein 90% of cell confluence was observed (visual observation), which suggests that the solvent (5% DMSO) did not have a toxicological effect.

Previously, Kenaf seed-mediated silver nanoparticles showed promising anti-lung cancer activity [15]. It was reported that the plant possesses bioactive compounds which induce mitochondrial damage by elevating the superoxide level that suppresses cancer cell growth through reduction of ATP synthesis [40]. Some secondary metabolites may also attack the DNA, resulting in abundant ROS production and apoptosis-inducing enzyme activation, subsequently leading to cell death [41].

### 4.6. Antibacterial Activity

The antibacterial activity of Kenaf leaves and seed extracts are presented in Table 2. Both Gram-positive (*Staphylococcus aureus*, *Bacillus cereus*, and *Bacillus subtilis*) and Gram-negative (*Salmonella Typhi*, *Escherichia coli*, and *Pseudomonas aeruginosa*) microorganisms were tested through the disc diffusion method. Results revealed that Kenaf seed extracts were very effective against both Gram-positive and Gram-negative bacteria. Ethyl acetate, ethanol, and water extract exposed antibacterial activity against *B. cereus*, *E. coli*, and *B. subtilis* microorganisms. Here, the most remarkable inhibitory effect was observed against *E. coli* bacteria, where the zone of inhibition was recorded for 15.2 ± 0.72 mm for ethyl acetate extract. In the case of leaf extracts, the zone of inhibition recorded for ETH and WT were the most potent among all other extracts. These results indicate that the Kenaf plant possesses a broad spectrum antipathogenic effect. Moreover, both leaves and seed extracts of NHX did not show any inhibitory effect against any bacteria. On the other hand, both leaf and seed extracts did not manifest any activity against Gram-positive (*Staphylococcus aureus*) and Gram-negative (*Salmonella Typhi* and *Pseudomonas aeruginosa*) microorganisms. In the case of the negative control (5% DMSO), no activity was recorded, which indicates that the 5% DMSO did not have an influence on the extract’s activity.

The observed antibacterial efficiencies of the Kenaf plant might be due to the higher content of phenols and flavonoids [42]. It is stated that the secondary metabolites present in the plant are the key source of diverse pharmacological actions [43]. These metabolites also provide natural defensive pathways to inhibit various insects and pathogens, such as viruses and fungi [44]. In addition, GC-MS analysis of Kenaf leaves and seed exposed several potential bioactive constituents, which might play a crucial role either by interrupting normal cellular functions [45] or destabilizing the bacterial membrane [46].

## 5. Conclusions

This research explored the various solvent extractions of Kenaf leaves and seed and their pharmacological potentials. Among the solvents tested, ethanol was the efficient solvent, and demonstrated the highest extraction yield and potential bioactive compounds. In contrast, the water solvent remarkably influenced phytochemical content. In the case of leaves and seed, water extracts significantly impacted on the recovery of the highest total polyphenol, flavonoids, and manifestation of noteworthy antioxidant activity. In vitro cytotoxic activity revealed that extracts of Kenaf leaves and seed were non-toxic to the healthy (NIH3T3) cells, except *n*-hexane extracts, which expressed slight toxicity. Besides, *n*-hexane and ethanol extracts manifested promising anti-lung cancer activity against A549 cells, where ethanol extracts were comparatively safer than other extracts. During the antibacterial test, extracts of Kenaf seed exhibited significant activity against Gram-positive and Gram-negative microorganisms, as evidenced by the notable zone of inhibition. This work collectively concluded that Kenaf seed extracts was found very potent in terms of important bioactive compounds and pharmacological aspects, which can be an excellent biological matrix of natural antioxidants.

## Figures and Tables

**Table 1:** List of compounds identified in Kenaf leaves and seed ethanol extract obtained by GC-MS analysis.

S/L no	R. Time (min)	PA (%)	Compound Name	Molecular Formula	MW (g/mol)	*** Activity
**Leaves**						
1	3.42	1.56	2-(Tert-butylamino)-3-methyl-2-pentenenitrile	C_10_H_18_N_2_	166.3	No activity
2	3.51	1.25	5-Methylfurfural	C_6_H_6_O_2_	110.1	Antioxidant, antiproliferative, antibacterial
3	4.01	1.75	Cyclopropanecarboxamide	C_4_H_7_NO	85.1	No activity
4	4.24	2.36	5-Amino-6-nitrosopyrimidine-2,4(1h,3h)-dione	C_4_H_4_N_4_O_3_	156.1	Antibacterial
5	4.47	0.25	Hexyl octanoate	C_14_H_28_O_2_	228.4	Flavouring agent
6	4.54	1.43	Pyrrolidin-5-one, 2,3-dedihydro-3-nitro-	C_4_H_3_NO_4_	129.1	No activity
7	4.69	0.27	3-Amino-2-oxazolidinone	C_3_H_6_N_2_O_2_	102.1	No activity
8	4.76	3.70	4H-Pyran-4-one, 2,3-dihydro-3,5-dihydroxy-6-methyl-	C_6_H_8_O_4_	144.1	Antibacterial, anti-inflammatory, antiproliferative antioxidant, automatic nerve activity, anticancer
9	5.13	0.84	3,4-Pentadienal	C_5_H_6_O	82.1	No activity
10	5.27	0.34	cyclobut-1-enylmethanol	C_5_H_8_O	84.1	Antibacterial
11	5.32	7.20	5-Hydroxymethylfurfural	C_6_H_6_O_3_	126.1	Anti-oxidative, anti-allergic, anti-inflammatory, anti-hypoxic, anti-hyperuricemic
12	5.89	0.21	6-O-Acetyl-beta-D-glucose-	C_8_H_14_O_7_	222.2	
13	5.93	0.43	6-Oxa-1-azabicyclo(3.1.0)hexane, 2,2-dimethyl-4,5-diphenyl-, trans-	C_18_H_19_NO	265.3	Antibacterial
14	6.12	0.62	N-(2-Methoxyethyl)alanine	C_6_H_13_NO_3_	147.2	No activity
15	6.17	1.00	trans-2-Butenyl acetate	C_6_H_10_O_2_		No activity
16	6.81	0.96	N-Acetyl-d-serine	C_5_H_9_NO_4_	147.1	No activity
17	6.84	1.03	alpha-D-Mannopyranoside, methyl 3,6-anhydro-	C_7_H_12_O_5_	176.2	Antibacterial
18	7.19	0.27	2(4H)-Benzofuranone, 5,6,7,7a-tetrahydro-6-hydroxy-4,4,7a-trimethyl-	C_11_H_16_O_3_	196.2	Antibacterial
19	7.24	0.46	2-Mercaptopyridine-4-ol	C_5_H_5_NOS	127.17	No activity
20	7.71	3.5	1,3,4,5-Tetrahydroxycyclohexanecarboxylic acid	C_7_H_12_O_6_	192.2	Hepatoprotective
21	8.15	1.00	d-Glycero-d-galacto-heptose	C_7_H_14_O_7_	210.1	Antioxidant
22	8.27	0.30	7-Hydroxy-3-(1,1-dimethylprop-2-enyl)coumarin	C_14_H_14_O_3_	230.3	Antibacterial, antitumor
23	8.30	1.11	N-Acetyl-D-Glucosamine	C_8_H_15_NO_6_	221.1	Antibacterial, antitumor, antioxidant, anticoagulant, wound healing
24	8.39	0.64	Z-8-Methyl-9-tetradecen-1-ol acetate	C_17_H_32_O_2_	268.4	No activity
25	8.5	0.21	3-Methyl-4-(phenylthio)-2-prop-2-enyl-2,5-dihydrothiophene 1,1-dioxide	C_14_H_16_O_2_S_2_	280.4	No activity
26	8.75	0.69	Methyl tricosanoate	C_24_H_48_O_2_	368.6	Antibacterial
27	8.93	2.05	Palmitic acid	C1_6_H_32_O_2_	256.4	Antioxidant, antitumor, anti-inflammatory, antibacterial, antifungal
28	9.14	0.77	Pentadecanoic acid	C_15_H_30_O_2_	242.4	Adhesives, agricultural chemicals (non-pesticidal), lubricants
29	9.19	0.41	26-Hydroxycholesterol	C_27_H_46_O_2_	402.7	No activity
30	9.26	0.50	Hexacosanoic acid	C2_6_H_52_O_2_	396.7	No activity
31	9.32	0.60	Palmitic acid	C1_6_H_32_O_2_	256.4	Antioxidant, antitumor, anti-inflammatory, antibacterial, antifungal
32	9.43	1.78	9,12,15-Octadecatrienoic acid, methyl ester	C_16_H_32_O_2_	292.5	No activity
33	9.47	1.24	Phytol	C_20_H_40_O	296.5	Antioxidant, analgesic, antibacterial, anti-inflammatory, anticancer, and neuroprotective
34	9.61	2.96	1,5,9,13-Tetradecatetraene	C_14_H_22_	190.3	No activity
35	9.80	3.40	2-Linoleoylglycerol	C_21_H_38_O_4_	354.5	Antibacterial
36	9.94	0.42	Stearic acid	C_18_H_36_O_2_	284.5	Antioxidant, antibacterial
37	9.98	0.50	Epoxycholesterol	C_27_H_46_O_2_	402.7	No activity
38	10.06	3.06	Hexadecanoate	C_16_H_31_O_2_	255.42	Anti-inflammatory
39	10.21	1.56	4alpha,5alpha-Epoxycholestane	C_27_H_46_O	386.7	No activity
40	10.70	2.83	6,10,14-Trimethylpentadecan-2-one	C_18_H_36_O	268.5	antibacterial
41	10.80	1.05	1-Cinnamyl-3-methylindole-2-carbaldehyde	C_19_H_17_NO	275.1	Antioxidant, antibacterial
42	10.85	0.92	3,5-Bis-(trichloromethyl)-benzoyl chloride	C_9_H_3_Cl_7_O	375.3	No activity
43	10.92	5.44	2-Stearoylglycerol	C_21_H_42_O_4_	358.6	No activity
44	11.60	1.58	Tricyclo[10.2.2.2(5,8)]octadeca-5,7,12,14,15,17-hexaene	C_18_H_20_	236.3	Antifungal
45	11.70	2.17	9,12,15-Octadecatrien-1-ol	C_18_H_32_O	264.4	Antioxidant, antibacterial
46	11.74	4.50	1,5,9,13-Tetradecatetraene	C_14_H_22_	190.3	No activity
47	11.79	3.16	5 beta-Coprostanol	C_27_H_48_O	388.7	No activity
48	12.24	1.64	Curan-17-oic acid, 2,16-didehydro-20-hydroxy-19-oxo-, methyl ester	C_20_H_22_N_2_O_4_	354.4	Antibacterial, antifungal
49	14.65	4.45	Vitamin E	C_29_H_50_O_2_	430.7	Antioxidant, antibacterial, Analgesic, anti-inflammatory, anxiolytic and antidepressant,
50	15.28	1.52	3-{[(3,5-Dichlorophenyl)imino]methyl}-1,2-benzenediol	C_13_H_9_Cl_2_NO_2_	282.1	Antibacterial, antifungal
51	16.05	0.85	Methyl 4-oxo-4,5,6,7-tetrahydro-1H-indole-2-carboxylate	C_10_H_11_NO_3_	193.2	Antidiabetic
52	16.26	1.07	Stigmasta-5,22-dien-3-ol	C_29_H_48_O	412.7	Antibacterial
53	17.05	3.93	Clionasterol	C_29_H_50_O	414.7	Antibacterial
54	17.81	1.71	Hexadecahydropyrene	C_16_H_26_	218.3	No activity
55	18.56	3.76	alpha-Amyrin	C_30_H_50_O	426.7	Analgesic, anti-inflammatory, anxiolytic and antidepressant
**Seed**						
1	4.00	0.92	Glycerine	C_3_H_8_O_3_	92.1	Antibacterial
2	5.29	0.03	Benzofuran, 2, 3-dihydro-	C_15_H_14_OS	242.3	Antidepressant, anticancer, antiviral, antifungal, antioxidant, anti-psychotic
3	6.89	0.16	N-Acetylethylenediamine	C_4_H_10_NO_2_	102.1	Antibacterial
4	7.76	0.08	d-Mannitol, 1, 4-anhydro-	C_6_H_12_O_5_	164.2	No activity
5	8.08	0.03	Octadecanoic acid	C_18_H_36_O_2_	284.5	Antibacterial
6	8.75	0.05	Hexadecanoic acid, methyl ester	C_17_H_34_O_2_	270.5	Antibacterial, anticancer, anti-inflammatory, anti-diuretic
7	9.05	8.83	Hexadecanoic acid	C_16_H_32_O_2_	256.4	Antioxidant, antitumor, anti-inflammatory, antibacterial, antifungal
8	9.43	1.42	Hexadecanoic acid	C_16_H_32_O_2_	256.4	Antioxidant, antitumor, anti-inflammatory, antibacterial, antifungal
9	9.81	77.46	9-octadecanoic acid (z)-	C_18_H_34_O_2_	282.5	Antibacterial, antifungal
10	10.71	1.58	Linoleic acid	C_18_H_32_O_2_	280.4	Antibacterial
11	10.95	2.85	Linoleic acid	C_18_H_32_O_2_	280.4	Antibacterial
12	11.75	6.21	9, 12-octadecadienoic acid (z, z)-, 2-hydroxy-1-(hydroxymethyl)ethyl ester	C_21_H_38_O_4_	354.5	Antioxidant, antibacterial
13	13.97	0.33	17-(acetyloxy)-4, 4-dimethyl-7-oxoandrost-5-en-3-yl acetate	C_25_H_36_O_5_	416.5	No activity
14	17.07	0.04	.beta.-Sitosterol	C_29_H_50_O	414.7	Antidiabetic, antibacterial

SL no: serial number, R.T: retention time, PA: peak area, MW: molecular weight, *** Activity Source: Dr. Duke’s phytochemical and ethno-botanical databases [30].

**Table 2:** Antibacterial effects of various solvent extracts of the Kenaf leaf and seed.

			Zone of Inhibition (mm)			
Bacterial Strain	Gram Negative		Gram Positive			
Name of Bacteria	*Escherichia Coli*		*Bacillus Cereus*		*Bacillus Subtilis*	
Concentration (50 mg/mL)	Leaves	Seed	Leaves	Seed	Leaves	Seed
NHX	-	-	-	-	-	-
EA	-	15.2 ± 0.72	-	12.8 ± 0.78	-	11.6 ± 0.59
ETH	9.8 ± 1.41	13.1 ± 0.34	-	11.2 ± 0.37	-	10.1 ± 0.41
WT	11.3 ± 0.38	13.7 ± 0.56	9.7 ± 0.73	12.5 ± 0.82	-	9.3 ± 0.28
Standard Ampicillin (25 ug/mL)	29.4 ± 0.26	27.9 ± 0.48	26.4 ± 0.52	28.4 ± 0.47	30.1 ± 0.18	28.4 ± 0.47

Values are presented as mean inhibition zone (mm) ± SD of three replicates, -: no activity, NHX = *n*-Hexane extract, EA = Ethyl acetate extract, and WT = Water extract.
